# Development and Characterization of Genomic and Expressed SSRs in Citrus by Genome-Wide Analysis

**DOI:** 10.1371/journal.pone.0075149

**Published:** 2013-10-28

**Authors:** Sheng-Rui Liu, Wen-Yang Li, Dang Long, Chun-Gen Hu, Jin-Zhi Zhang

**Affiliations:** Key Laboratory of Horticultural Plant Biology (Ministry of Education), College of Horticulture and Forestry Science, Huazhong Agricultural University, Wuhan, China; Wuhan University, China

## Abstract

Microsatellites or simple sequence repeats (SSRs) are one of the most popular sources of genetic markers and play a significant role in plant genetics and breeding. In this study, we identified citrus SSRs in the genome of Clementine mandarin and analyzed their frequency and distribution in different genomic regions. A total of 80,708 SSRs were detected in the genome with an overall density of 268 SSRs/Mb. While di-nucleotide repeats were the most frequent microsatellites in genomic DNA sequence, tetra-nucleotides, which had more repeat units than any other SSR types, had the highest cumulative sequence length. We identified 6,834 transcripts as containing 8,989 SSRs in 33,929 Clementine mandarin transcripts, among which, tri-nucleotide motifs (36.0%) were the most common, followed by di-nucleotide (26.9%) and hexa-nucleotide motifs (15.1%). The motif AG (16.7%) was most abundant among these SSRs, while motifs AAG (6.6%), AAT (5.0%), and TAG (2.2%) were most common among tri-nucleotides. Functional categorization of transcripts containing SSRs revealed that 5,879 (86.0%) of such transcripts had homology with known proteins, GO and KEGG annotation revealed that transcripts containing SSRs were those implicated in diverse biological processes in plants, including binding, development, transcription, and protein degradation. When 27 genomic and 78 randomly selected SSRs were tested on Clementine mandarin, 95 SSRs revealed polymorphism. These 95 SSRs were further deployed on 18 genotypes of the three generas of Rutaceae for the genetic diversity assessment, genomic SSRs generally show low transferability in comparison to SSRs developed from expressed sequences. These transcript-markers identified in our study may provide a valuable genetic and genomic tool for further genetic research and varietal development in citrus, such as diversity study, QTL mapping, molecular breeding, comparative mapping and other genetic analyses.

## Introduction

Conventional plant breeding is largely dependent on selection of desirable plants which is highly decided by the parents and different environment factors interaction [1]. During conventional plant breeding genes are mixed and newly assorted. This results in non-desired traits being inherited together with the trait of interest. Thus, to develop a new variety, it may take 10–15 years especially for woody plants [2]. Marker-assisted selection (MAS) can be performed on seedling material, thus reducing the time required. On the other hand, MAS is not affected by environment condition [3]. Therefore, marker-assisted selection has become popular in breeding program in many crops in recent years [4], [5]. The development of high-density genetic linkage maps and subsequent quantitative trait locus (QTL) mapping of agronomically important traits is one of the prerequisites for employing MAS. Molecular markers are useful tools for development of genetic linkage maps and quantitative trait locus (QTL) mapping. Thus, the progress made in the development of DNA based marker systems has advanced including restriction fragment length polymorphisms (RFLPs), random amplification of polymorphic DNAs (RAPDs), amplified fragment length polymorphisms (AFLPs) and simple sequence repeats (SSRs) [6]. Among all these, SSRs are highly versatile, PCR-based markers, usually associated with a high frequency of length polymorphism, and thus useful in the development of high-density genetic maps, gene mapping and MAS. SSRs, also known as microsatellites, are tandemly repeated DNA sequences which are tandem repeats of 1 to 6 nucleotide long DNA motifs, have gained considerable importance in plant genetics and breeding because of their multi-allelic nature, reproducibility, co-dominant inheritance, high abundance and extensive genome coverage [6], [7]. They are ubiquitous in prokaryotes and eukaryotes, even in the small virus genomes [8], and they can be found in both protein coding and non-coding regions affecting gene expression [9]. During the last decade, microsatellites have proven to be the marker of choice in plant genetics and breeding research, because of their variability, ease of use, accessibility of detection and reproducibility [10]. There are now many well-known examples of initiatives using microsatellites for different plant species [11]–[14].

An innovative marker system that has been developed links expressed sequence tags (ESTs) and SSRs [12]. EST-SSRs have been found to be significantly more transferable across taxonomic boundaries [15] and more conserved than genomic SSRs [16]. Thus, these EST-SSRs have been applied successfully in studies of genetic variation, linkage mapping, comparative mapping, functional diversity analysis, evolution and sequencing of several plant genomes [17]. For the conventional strategies for SSR isolation, i.e., screening small insert genomic DNA libraries or constructing SSR-enriched libraries [10], are labor-intensive and time-consuming. However, the release of the genome sequence will dramatically enhance the efficiency of the SSR survey for an organism, because predicted transcripts are available based on completely genome. Transcript sequences constitute a rich and special source of informative molecular markers because they represent genes that are expressed in an organism. Coding sequences are generally more informative than anonymous markers because they allow for a more direct association between the molecular marker and the phenotype.

Citrus is a woody perennial tree grown around the world for the production of fresh fruit and juice amongst other products. In addition to its agronomical and economical importance, the citrus industry has a great social and cultural interest as part of our heritage. Although some SSRs were identified based on EST database in previous studies [13], [14], no system analysis of SSRs in citrus has been reported because of incomplete citrus genome. Recently, the Clementine mandarin genome has been sequenced [18], and the completion of these genome sequences provided an opportunity for us to scan the entire genome for SSR discovery in citrus. In this study, we present our results on the SSR survey for the development of citrus SSR markers. The survey was based on genome data, their classification, characterization and comparative analysis in eighteen phylogenetically distant citrus species including deciduous and evergreen citrus trees. This study will serve as reference for future comparative mapping studies and for the development of strategies that take advantage of DNA sequence analyses for cross-referencing genes between species and perhaps genera.

## Materials and Methods

### Identification of microsatellites and primer design

Genomic, transcript and CDS sequences of Clementine mandarin were downloaded from the Phytozome database (www.phytozome.net/). The Perl script MIcroSAtelitte (MISA) was used to identify microsatellites in all these genomes (http://pgrc.ipk-gatersleben.de/misa/). To identify the presence of SSRs, only 2 to 6 nucleotides motifs were considered, and the minimum repeat unit was defined as 6 for di-nucleotides, 5 for tri-nucleotides, 4 for tetra-nucleotides, and 3 for penta-nucleotides and hexa-nucleotides. Compound SSRs were defined as ≥2 SSRs interrupted by ≤100 bases [19].

The SSR information generated by MISA was used for designing primers flanking the repeats. To design primers flanking the microsatellite loci, two perl scripts were used as interface modules for the program-to-program data interchange between MISA and the primer designing software Primer 5. The SSR-containing transcripts were then identified as candidates for SSR marker development if they had sufficient sequences on both sides of the SSR repeats for primer design. Primer 5 software was used to design the primers. The following parameters were used: primer length 18–24 bp, with 20 bp as the optimum; primer GC%  = 40–70%, with the optimum value being 50%; primer Tm 50–60°C, and product size range 100–500 bp.

### SSR screening and polymorphism survey

For primer validation, plant samples were obtained from the germplasm collection of trifoliate orange, precocious trifoliate orange, Fenghuang pummelo, grape fruit, sweet orange, Clementine mandarin, lchang papeda, Newhall Navel Orange, Papeda, Sour orange, Guoqing no.1 Satsuma, Bendizaoju mandarin, Fortunella, Kumquat, HongJu tangerine, Calamondin, lemon and citron. All the eighteen citrus samples were collected in the experiment fields of the National Citrus Breeding Center at Huazhong Agricultural University (30°28′ N, 114°21′ E, 30 m). Total DNA was isolated from the leaf according to the cetyltrimethyl ammonium bromide method [20]. One hundred and five primers were randomly chosen and synthesized by Invitrogen, Shanghai PR China. PCR amplification was conducted in 25 µl reactions containing 50 ng of template DNA, 2.5 μM MgCl2, 2.5 μl 10×PCR buffer, 0.5 mM each primer, 0.5 U Taq DNA polymerase, and 2.5 mM dNTPs. The PCR cycling profile was 94°C for 5 min, 35 cycles at 94°C for 45 s, 58°C for 45 s, 72°C for 45 s, and a final extension at 72°C for 10 min. The quality of the PCR product was checked by mixing it with an equal volume of loading buffer and then visualizing the band on a 2.0% agarose gel in TBE buffer at 100 W for 120 min.

### Functional assignments of the transcripts containing SSRs

To assign putative functions to the SSR-containing transcripts, Blast2go program was run locally to BLAST against a reference database that stores UniProt entries, their associated Gene Ontology (GO), Enzyme Commission (EC), and Kyoto Encyclopaedia of Genes and Genomes (KEGG) annotation [21], the GO categorization results were expressed as three independent hierarchies for biological process, cellular component, and molecular function.

### Genetic diversity and data analysis

POPgene (v1.32) was used to calculate the different statistical and genetic parameters [22] such as the effective allele number [23] and the Shannon's Information index [24], expected heterozygosity [25], [26]. The Genetic distance of the SSRs genotype was calculated based on the Nei's genetic distance measure with Pairwise distance calculation by MEGA 4.1. Polymorphic information content (PIC) value for SSR marker was calculated following [27]. A dendrogram was constructed based on unweighted pair-group method with arithmetic mean (UPGMA) by MEGA4.

## Results

### Diversity of SSRs in the Clementine mandarin genome

**Figure 1:**
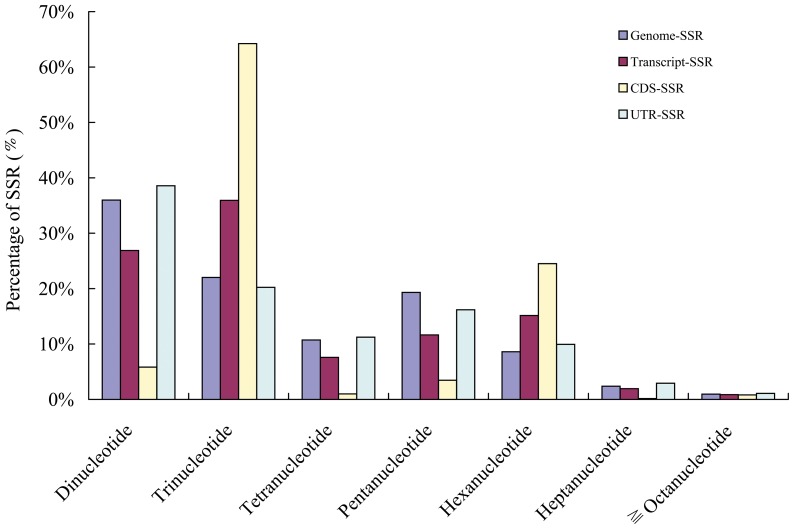
SSR frequency according to estimated location (Genome, transcript, CDS and UTR).

A total of 80,708 microsatellites, consisting of a variety of repeat types, were identified in the Clementine mandarin genome (v182) assembly by MISA (Table S1). The genome average SSR density was approximately 268 per mega-base (MB). Di-nucleotide repeats were the most abundant, accounting for 36.0% (29,041) of all SSRs. Tri-, penta-, tetra-, and hexa-nucleotide repeats accounted for 22.0% (17,773), 19.3% (15,595), 10.7% (8,659), and 8.6% (6,939), respectively, of all SSRs (Figure 1). Of the di-nucleotide repeats, AT/TA was the most abundant, accounting for 51.8% (15,057) of all di-nucleotide repeats, while AG/CT and AC/GT repeats accounted for 26.4% (7,673) and 21.1% (6,137), respectively. It is worth noting that SSR motifs represented variants of both strands of the DNA sequence. CG/GC repeats were rather rare (0.6%, 174), and only 56 CG repeats were found. Among tri-nucleotide repeats, AAT/TTA was the most abundant, accounting for 49.5% (8,804) of all tri-nucleotide repeats, followed by AAG/CTT (9.2%, 1,604) and ATC/TAG (3.3%, 591). Of tetra-nucleotide repeats, AAAT/TAAA was the most abundant, accounting for 34.8% (3,017) of all tetra-nucleotide repeats, and followed by ACAT/ATGT (18.5%, 1,078). Among penta- and hexa-nucleotide repeats, AT-rich repeats were the most abundant, accounting for 62.1% (9,680) and 32.2% (2,232) of all penta- and hexa-nucleotide repeats, respectively. Moreover, 89.4%, 91.0%, 97.2%, 99.3%, and 99.3% of di-, tri-, tetra-, penta-, and hexa-nucleotide repeats, respectively, were less than 30 bp in length. A small number of di-nucleotide repeats (2.0%) were longer than 50 bp in length, whereas few tri-, tetra-, pentra-, or hexa-nucleotide repeats (<1%) were longer than 40 bp in length.

We examined the distribution of Clementine mandarin microsatellites with regard to the number of repeat units. For all SSR classes, microsatellite frequency decreased as the number of repeat units increased. However, the rate of this change was more gradual in di-nucleotides than in longer repeat types, with tetra- to octa-nucleotides showing the most dramatic reduction in frequency as they increased repeat units. As a consequence, the mean number of repeat units in di-nucleotides (9.4) was nearly 1.5 times as much as the number of repeat units in tri-nucleotides (6.6) and it was twice higher than in tetra-nucleotides (5.5). Thus, di-nucleotides (96.5 SSRs/Mb) occurred more frequently than tetra-nucleotides (59.0 SSRs/Mb) in the Clementine mandarin genome, the di-nucleotide repeats, due to their higher number of repeat units, had a greater contribution to the genome fraction occupied by SSRs: the cumulative sequence length of di- and tetra-nucleotide repeats was 275.5 kb and 118.1 kb, respectively.

### Frequency and distribution of transcript-SSRs in the transcriptome

In order to isolate transcript-SSRs from citrus, a total of 33,929 transcripts were downloaded from Clementine mandarin genome with a total length of 56.5 Mb and an average length of 1707.6 bp ranging from 99 bp to 15,710 bp. Transcribed regions of the Clementine mandarin genome had fewer microsatellites compared to its genomic sequence, a total of 6,834 sequences containing 8,989 SSRs have been identified from these transcripts, with 1,581 sequences containing at less two SSRs (Table S2). The frequency of transcript-SSRs observed in Clementine mandarin transcriptome was 20.1%, and the distribution density was 159.1 per Mb. The most abundant repeat type was tri-nucleotide (36.0%, 3,230) followed by di- (26.9%, 2,417), hexa- (15.1%, 1,361), penta- (11.6%, 1,047), tetra-nucleotide (7.6%, 682) and other repeat unit (2.8%, 252) (Table 1).

**Table 1:** Frequency of different type of motif in Di-, Tri-, Tetra-, Penta-, Hexa-, and other-nucleotide (> hexanucleotide) SSRs from Clementine mandarin.

Motif length	location	Repeat number									Total	Total (%)
		3	4	5	6	7	8	9	10	>10		
Di	Genome	-	-	-	7,806	4,966	3,763	2,823	2,257	7,426	29,041	36.0
	Transcript	-	-	-	747	382	312	282	178	514	2,417	26.9
	CDS	-	-	-	99	18	11	26	6	27	187	5.8
	UTR	-	-	-	648	364	301	256	172	487	2,228	38.5
Tri	Genome	-	-	8,248	4,027	2,068	1,187	647	402	1,194	17,773	22.0
	Transcript	-	-	1,585	819	395	213	107	45	66	3,230	35.9
	CDS	-	-	1,049	529	245	125	59	28	25	2,060	64.2
	UTR	-	-	536	290	150	88	48	17	41	1,170	20.0
Tetra	Genome	-	5,974	1,834	437	175	76	68	44	51	8,659	10.7
	Transcript	-	449	150	59	14	5	3	2	0	682	7.6
	CDS	-	21	9	2	0	0	0	0	0	32	1.0
	UTR	-	428	141	57	14	5	3	2	0	650	11.2
Penta	Genome	12,760	2,346	382	76	17	10	2	0	2	15,595	19.3
	Transcript	825	163	45	10	4	0	0	0	0	1,047	11.6
	CDS	102	6	1	1	1	0	0	0	0	111	3.5
	UTR	723	157	44	9	3	0	0	0	0	936	16.1
Hexa	Genome	5,869	872	153	28	13	1	1	1	1	6,939	8.6
	Transcript	1,095	211	44	9	0	0	1	1	0	1,361	15.1
	CDS	623	128	25	8	0	0	1	1	0	786	24.5
	UTR	472	83	19	1	0	0	0	0	0	575	10.0
Others	Genome	2,243	296	76	23	22	10	6	2	23	2,701	3.3
	Transcript	214	22	8	3	2	3	0	0	0	252	2.8
	CDS	21	9	1	0	0	0	0	0	0	31	1.0
	UTR	193	13	7	3	2	3	0	0	0	221	3.8

The distribution of transcript-SSRs with various motifs was also studied across different repeat numbers. Our results show that the distribution of all di-, tri-, tetra-, penta-, and hexa-nucleotide transcript-SSRs was skewed generally to the smaller number of repeats. A few higher repeat numbers were observed in di- and tri-nucleotide SSRs, but in tetra-, penta-, and hexa-nucleotide SSRs, no repeat number was found beyond 6 (Table 1). The average number of repeats transcript-SSR markers in Clementine mandarin was 5.7 repeats per SSR with the range from the low average number (3.2) in penta-nucleotide motifs to the high average number (8.1) in di-nucleotide motifs. The frequencies of transcript-SSRs distributed in different repeat numbers were shown in Table 1. For example, there were 2,134 SSRs with 3 tandem repeats, which wasthe most common repeat number (23.7%) followed by 5 tandem repeats (20.6%, 1,852), 6 tandem repeats (18.3%, 1,647), 7 tandem repeats (9.4%, 845) and 4 tandem repeats (8.9%, 797). The longest number of repeats was observed in TA motif, (TA)_37_ in transcripts (Ciclev10002043m and Ciclev10001815m) (Table S2). The dominant repeat motif was AG/CT with a frequency of 16.7% (1,504), followed by AAG/CTT (6.6%, 596), AT/TA (6.2%, 561), AAT/ATT (5.0%, 451), ATC/TAG (2.2%, 197) and AGC/TCG (2.1%, 193). It is interesting that there was no CG/GC repeat motif and very few CCG/CGG repeats in our results. More details about different repeat motif of di- and tri-nucleotide repeats in transcript-SSRs were listed in table S2. The greatest number of SSRs detected was seven in a transcript sequence (Ciclev10002043m) (Table S2).

### Distribution of microsatellites in the coding regions

Estimating the location of microsatellites within genes (coding, 5′ untranslated regions or 3′ untranslated regions) is important when using transcript-SSRs to study microsatellite evolution and in marker development. Among the 33,929 predicted protein-coding loci (CDS) searched, the total length of the coding region was 41.7 Mbp, representing 69.0% of the total transcript length. The average length of the coding region in each contig was 414.0 amino acids (aa), ranging from 33 to 5,179 aa. A total of 3,207 microsatellites were identified in 2,672 CDS, including 412 CDS containing at least two microsatellites (Table S3). They represent 35.7% of the total number of microsatellite loci found in the whole transcriptome. Overall, the distribution density of microsatellites was 76.9 per Mb of coding sequence. Only hex-, di- and tri-nucleotide motifs were observed, this last category representing more than 94.6% of the total set of microsatellites (Table 1). Because the addition or deletion of di-nucleotide repeats located within coding regions can cause frame shifts, selective pressures disfavour the presence of di-nucleotide in coding regions. We also examined the locations of specific SSR motifs. The most common motifs in the coding regions were AG and AC, respectively, with AG motifs accounting for 137 (73.3%) and AC motifs representing 32 (17.1%) of all di-nucleotide motifs in coding regions.

The 5′ untranslated regions (UTR) and 3′-UTR regions in Clementine mandarin contain a total of 5,782 SSRs. The average SSR density was approximately 393.3/MB, SSRs with periods of 2 to 10 account for 38.6% (2,230), 20.2% (1,170), 11.2% (650), 16.2% (936), 10.0% (575), 2.9% (169), 0.7% (41), 0.1% (7), and 0.0% (4), respectively (Table 1). The observed densities of di-, tri-, and tetra-nucleotide SSRs were much higher in UTR than those exon regions. AT was also the most common motif in the UTR, accounting for 70 (10.7%) of all di-nucleotide in this region, although it was much less common here than in the 3′ UTR. The most common tri-nucleotide in the coding, 3′ UTR and 5′ UTR regions were AAG, AAT and AGG, respectively.

### Functional annotation of transcripts containing SSRs

To investigate biological processes possibly regulated by 6,834 sequences containing SSRs, all transcript sequences containing SSRs were used in a search of homology for proteins in the NCBI database by the Blast approach. We detected 5,879 sequences (86.0%) as having homology with known proteins, while 756 (11.1%) were homologous to expressed, hypothetical or unknown proteins. The remaining 199 (2.9%) sequences did not possess homology with any known proteins. In addition, 321 sequences were annotated relating to transcription regulator activity belonging to 60 families. The MYB family was the most prevalent, followed by the AP2/EREBP and WRKY families, part of which might play roles in regulating development and metabolism. All these structural and regulatory genes were being cloned and functional studies are carrying out in our lab. Further studies such as molecular modification (or gene transformation) and metabolic engineering of enzymes were also in progress.

**Figure 2:**
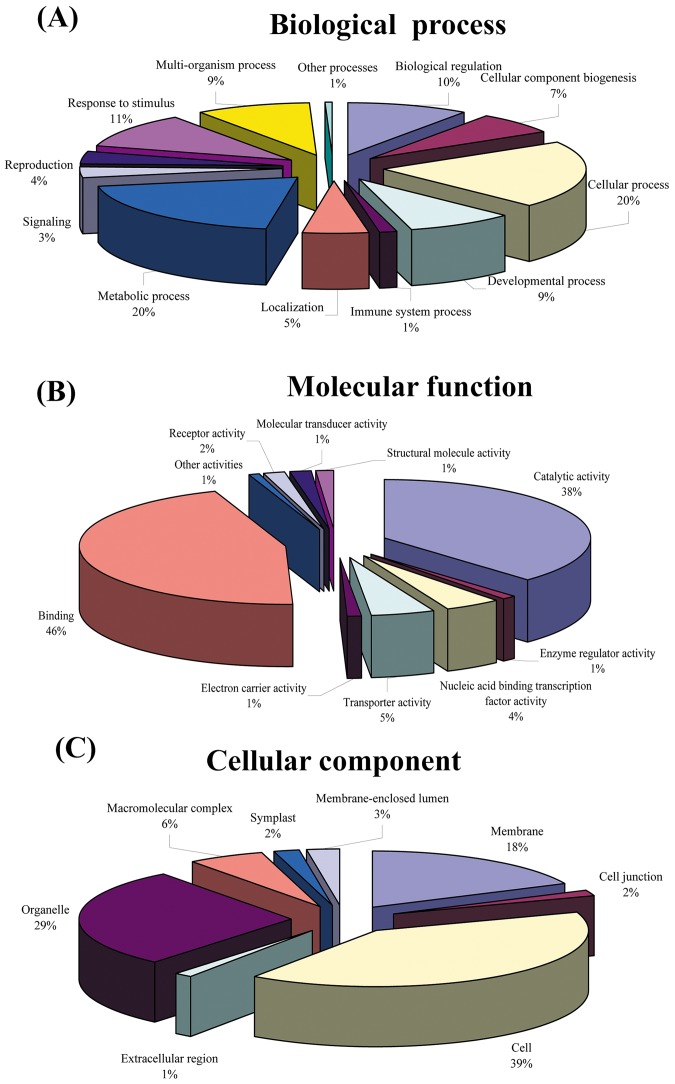
Characterization of Clementine mandarin 6, 834 sequences containing SSRs by gene ontology categories, A: Biological process; B: molecular function; C: cellular component.

**Figure 3:**
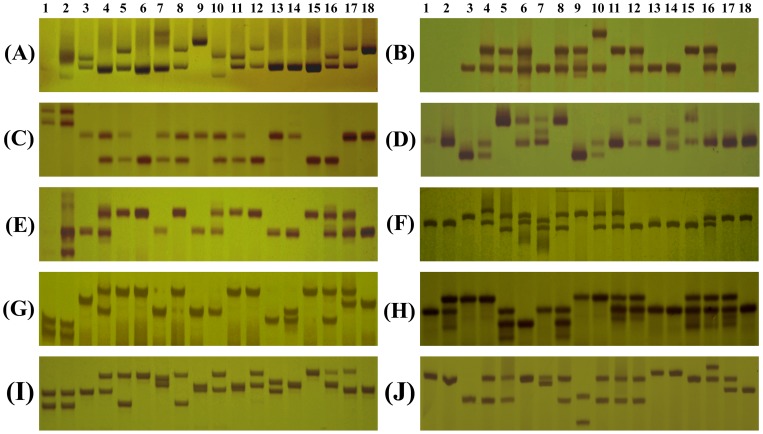
Polymorphism detected by ten SSR markers among 18 genotypes of three genera of Rutaceae.

GO annotation of these genes was performed by Blast2GO. Based on GO annotation, 91% (6,229 out of 6,834) sequences were able to be assigned GO numbers. A gene product might be associated with or located in one or more cellular components, such that it is active in one or more biological processes, during which it performs one or more molecular functions. Thus, some transcripts were annotated with the three categories simultaneously. Figure 2 showed the percentage distributions of gene ontology terms according to the GO consortium. Cellular process and metabolic process (20%) were the most dominant group out of 6,229 sequences that were annotated to the biological process category (Figure 2). It was followed by the response to stimulus at 11%, biological regulation at 10%, multi-organism process and developmental process at 9%, cellular component biogenesis at 7%, localization at 5%, reproduction at 4% and signaling at 3%. A total of 6,490 sequences could be assigned to the molecular function category (Figure 2). Binding (46%) was the most dominant group followed by catalytic activity (38%), transporter activity (5%), nucleic acid binding transcription factor activity (4%) and receptor activity (2%). The distribution of the sequences between specialized terms in the binding section of the molecular function category showed that the greatest numbers fell under small molecule binding (34.3%) and nucleic acid binding (36.7%). Interestingly, the third greatest number of the binding section fell into ion-binding (17%). With regard to the cellular component (Figure 3), 39% sequences were assigned to cell followed by organelle (29%), membrane (18%) and macromolecular complex (6%). The biological interpretation of the significant differential expression genes was further completed using KEGG pathway analyses. A total of 127 different pathways were found in this study, of which some were consistent with biological processes already revealed by GO analyses. The most represented pathways included purine metabolism (128 enzymes represented), starch and sucrose metabolism (96), pyrimidine metabolism (55), T cell receptor signaling pathway (43), glycolysis/gluconeogenesis (43) and glycerophospholipid metabolism (43). Of these, some were related with mutation trait formation based on previous knowledge, including biosynthesis of plant hormones, spliceosome, RNA degradation, ubiquitin mediated proteolysis, and calcium signaling pathway.

### The Clementine mandarin SSRs Transferability

A total of 105 primer pairs (27 genomic and 78 expressed SSRs) have been stochastically selected among the 80,708 microsatellites to amplify 25 di-, 37 tri-, 11 tetra-, 15 penta-, 17 hexa-nucleotide SSRs for testing amplification rate in Clementine mandarin (Table S4). Of these primer pairs, four primer pairs (one expressed SSR and three genomic SSRs) cannot amplify any fragment in Clementine mandarin, suggesting that these primers were not well designed. Six primers from expressed SSR amplify numerous non-target bands, suggesting these primers were also problematic (Table 2). Twenty-seven primer pairs (16 expressed SSRs and 11 genomic SSRs) succeed in Clementine mandarin, but amplify PCR products larger than the expected sizes. The rest of primers produced the expected size of amplicons in Clementine mandarin. Therefore, 90.5% (95 out of 105) primers from Clementine mandarin SSR can be amplified successfully. In theoretically, there were 64.8% (68/105) of 80,708, namely ∼52,268, Clementine mandarin SSR markers suitable for genetic studies in genus citrus.

**Table 2:** Cross-species transferability of Clementine mandarin derived genomic and expressed SSRs in three generas of Rutaceae.

Genera	species	Latin name	Ploidy	Cultivated/ Wild	No. of SSR amplified	% of SSR amplified	Absent of SSR amplified	No. of null amplified	PIC
Trifoliate orange	Trifoliate orange	*P. trifoliata*	2×	Wild	89	84.76	6	6	0.39
Trifoliate orange	Precocious trifoliate orange	*P. trifoliate*	2×	Wild	89	84.76	6	6	0.4
Citrus	Fenghuang pummelo	*C. grandis*	2×	Cultivated	93	88.57	3	5	0.42
Citrus	Grape fruit	*C. paradise*	2×	Cultivated	92	87.62	1	8	0.48
Citrus	Sweet orange	*C. sinensis*	2×	Cultivated	94	89.52	0	7	0.47
Citrus	Clementine mandarin	*C. clementina*	2×	Cultivated	93	88.57	1	7	0.45
Citrus	Yichang papeda	*C. ichangensis*	2×	Cultivated	95	90.48	0	6	0.44
Citrus	Newhall Navel Orange	*C. sinensis ‘Newhall’*	2×	Cultivated	94	89.52	1	6	0.48
Citrus	Papeda	*C. honghensis*	2×	Cultivated	93	88.57	1	7	0.4
Citrus	Sour orange	*C. aurantium*	2×	Cultivated	95	90.48	0	6	0.48
Citrus	Guoqing no.1 Satsuma	*C. unshiu*	2×	Cultivated	95	90.48	1	5	0.45
Citrus	Bendiguangju mandarin	*C. reticulata cv. Succosa*	2×	Cultivated	94	89.52	0	7	0.44
Fortunella	Fortunella Swingle	*Fortunella japonica*	2×	Cultivated	87	82.86	8	6	0.4
Fortunella	Kumquat	*Fortunella hindsii var. chintou*	2×	Cultivated	90	85.71	5	6	0.41
Citrus	HongJu tangerine	*C. reticulate ‘hongju’*	2×	Cultivated	94	89.52	0	7	0.44
Citrus	Calamondin	*C. reticulate ‘Calamondin’*	2×	Cultivated	92	87.62	0	9	0.46
Citrus	lemon	*C. limon*	2×	Cultivated	94	89.52	1	6	0.46
Citrus	Citron	*C. medica*	2×	Cultivated	90	85.71	5	6	0.38

To test cross-species/genera transferability 105 SSRs were tested on a panel of 18 citrus species including trifoliate orange, precocious trifoliate orange, Fenghuang pummelo, grape fruit, sweet orange, Clementine mandarin, lchang papeda, Newhall Navel Orange, Papeda, Sour orange, Guoqing no.1 Satsuma, Bendizaoju mandarin, Fortunella, Kumquat, HongJu tangerine, Calamondin, lemon and citron across three genus of Rutaceae (Table 2). The results indicated that the newly developed 95 SSRs from Clementine mandarin showed mean 84.5% amplification (89) in trifoliate orange genus, 84.5% (88.5) in fortunella swingle genera, and 88.9% (93) in citrus genera (Table 2). Furthermore, a high level of genetic diversity in 18 genotypes was discovered. The number of alleles per locus ranges from 2 to 9 with an average of 3.85 alleles. Ho ranges from 0 to 0.94 with an average of 0.32. He ranges from 0 to 0.86 with an average of 0.56. Polymorphism information content (PIC) was from 0.05 for Ciclev10001229m and Ciclev10024855m locus to 0.49 for Ciclev10001989m locus. The mean PIC value for Citrus genera was 0.45 (range 0.40–0.48), which was higher than 0.40 (range 0.39–0.40) and 0.42 (range 0.38–0.46) observed for trifoliate orange genera and fortunella genera, respectively. The above mentioned SSR allelic data, when used to calculate the heterozygosity estimates, revealed highly significant differences between the observed and expected heterozygosity for Citrus (mean Ho: 2.28 and mean He: 0.86), fortunella genera (mean Ho: 1.41; mean He: 0.33) and trifoliate orange genera (mean Ho: 1.23; mean He: 0.16). Thus, the results suggested significant heterozygote deficiency among the three genera in Rutaceae.

### The genetic diversity and dendrogram in citrus genus

**Figure 4:**
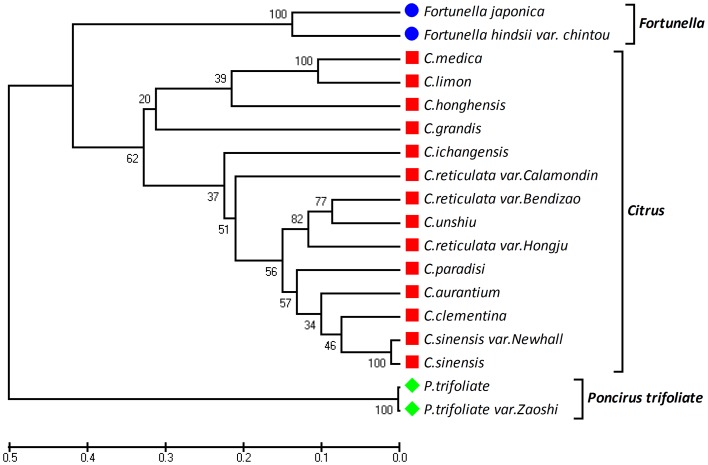
UPGMA tree of 18 genotypes of three genera of Rutaceae based on Nei's genetic distance using 95 SSRs.

Despite a relatively low level of polymorphism, the 25 genomic SSRs and 70 expressed SSRs data were also examined for their potential use in genetic diversity analysis among 18 cultivated genotypes of citrus (Figure 4). The UPGMA phylogenetic tree generated revealed three major clusters including two, two, fourteen genotypes in each clusters (trifoliate orange, fortunella and citrus), respectively. The UPGMA based clustering showed the grouping of all the two species of trifoliate orange in one (trifoliate orange cluster), two species of fortunella in one (fortunella cluster), and other fourteen species in one group (citrus cluster). In the citrus cluster, C. limon, *C. medica, C. grandis* and C. *honghensis* were more closely related and formed one sub-cluster than the other sub-cluster of citrus. The Nei's genetic distance [28] values ranged from 0.006 (*P. trifoliate* vs. *P. trifoliate var zaoshi*) to 0.475 (*P. trifoliate var zaoshi* vs. *C. sinensis Newhall*) with an average value of 0.332±0.085 (Figure 4). The out-group trifoliate orange and fortunella showed a relatively larger amount of average genetic distance (0.50 and 0.43) to all the genotypes of citrus (Figure 4).

## Discussion

The recent release of the Clementine mandarin genome sequence will dramatically enhance the efficiency of functional and comparative genomics research in citrus [18]. This allows researchers studying various agronomic traits related to the perennial trees with a completely new set of tools, including a potentially useful resource for mining SSR markers. In this study, the distribution and frequency of SSRs has been analyzed, with repeat unit lengths of 2 to 6 bp and a minimum length of 12 bp in the Clementine mandarin genome. A total of 80,708 SSRs were identified in the Clementine mandarin genome (Table S1). Given the estimated 301 Mb size of the Clementine mandarin genome [8], the SSR density was 268 per Mb in the DNA sequence of Clementine mandarin. This observed SSR frequency in the Clementine mandarin genome was lower than those reported for other plant species [29]. For example, overall densities of SSRs in genomes of *Arabidopsis*, rice, poplar, and grapevine are 370, 529, 508, and 506 SSRs/Mb, respectively. This was probably mainly due to the fact that higher stringent conditions have been used in defining SSRs in this study than those used previously for *Arabidopsis*, rice, a poplar, and grapevine [30]. A general negative correlation between genome size and SSR density in plants has been reported [29]. Our data in Clementine mandarin agrees with this general trend. In Clementine mandarin genomic DNA, microsatellites were present at a lower density than in coding regions, consistent with previous reports on soybean, rice and sorghum, which had 1.2 to 2.0-fold higher density in transcribed sequences than in genomic data.

On the other hand, we characterized also the SSRs in the entire genome sequencing assembly of Clementine mandarin and analyzed their frequency and distribution in different transcript regions. Most of the SSRs were di-nucleotides and tri-nucleotides, accounting for up to 89% of all of the SSRs identified. When examining the repeat number of the SSR motifs, our results showed that the distribution of all di-, tri-, tetra-, penta-, and hexa-nucleotide transcript-SSRs was skewed generally to the smaller number of repeats. We found that a small number of repeats (<10) were predominated. A few higher repeat numbers were observed in di- and tri-nucleotide SSRs, but in tetra-, penta-, and hexa-nucleotide SSRs, no repeat number was found beyond 6 (Table 1). Similar trends have also been observed in other plant genomes [31], [32]. The tri-nucleotide were predominant in Clementine mandarin, in agreement with other SSR survey studies [33] supporting the relative distribution of motifs in these plant groups. The most common di-nucleotide SSR motif was AG which comprised of 84.0% di-nucleotide motifs in all the Clementine mandarin transcripts. The motif AG was the most abundant and highly polymorphic in both annual and perennial plants including apple and citrus [34], [35]. However, this result was in inconsistent to previous findings in some species. Previous studies with genomic sequences showed that the most common di-nucleotide repeat was AT/TA in *Arabidopsis* and AC/CA in vertebrates and arthropods [12], [36]. The results may be attributable to the sources of the DNA sequences used (i.e. EST, cDNA, or gene sequences). Mun et al. (2006) have compared the frequency of motif AG in ESTs vs genomic sequences, and found that the higher frequency of motif AG in ESTs than in genomic sequences, for *Medicago truncatula*, *soybean*, *Lotus japonicus*, *Arabidopsis*, and rice [37]. The rarity of GC/CG repeats, observed in our analysis, seems to be common for all species and all genomic regions. For tri-nucleotide repeats, the most common repeat types varied among species as previously reported [38].

It has been noted that the SSRs in different locations within the gene might play different functional roles in organism development, adaptation, survival, and evolution were never-ending [39], [40]. For example, SSR expansions or contractions in protein coding regions and their UTRs can determine whether a gene becomes activated or inactivated by frameshift mutation or expanded toxic mRNA; intronic SSRs can affect gene transcription and mRNA splicing; SSR variations in 5′ UTRs could regulate gene expression and SSR expansions in 3′ UTRs may cause transcription slippage by affecting transcription and translation [39]. In Clementine mandarin, 3,087 SSRs were found in the CDSs of 2,402 genes, including many functional genes and transcription factors. The tri- and hexa-nucleotides in Clementine mandarin exhibited a higher density in CDSs. Such a propensity of tri- and hexa-nucleotides in the coding regions may be to suppress the other categories of SSRs, thus reducing the incidence of frame-shift mutations in coding regions caused by non-triplet repeats [41]. Total 5,742 SSRs were also found in the 5′ and 3′ UTRs of some genes coding for proteins (Table 1), these gene models were good candidates for further investigations. However, in general, SSRs in coding regions were less abundant than those in intergenic regions (Table 1). Our findings were in agreement with the fact that the majority of SSRs were embedded in non-coding DNA, either in the intergenic sequences or introns [42]. In addition, the AG rich content existing CDS-SSRs and the role of these motifs in the function of genes containing SSRs needs to be further investigated.

Although the role of the SSR motif in the function of plant genes was poorly understood, there was evidence showing that variation in the number of GA or CT repeats in the 5′ UTR of the waxy gene was related to the amylase content in rice [43] and motif CCG in 5′ UTR in ribosomal protein genes involved in the regulation of fertilization in maize [44]. Thus, putative functional annotation and categorization of transcript sequences containing SSRs in this study revealed that these sequences may be involved in various aspects of citrus development. Regarding biological processes, the majority of SSR loci found were involved with metabolic and cellular processes by comparing all transcript sequences with Gene Ontology assignment and those containing SSRs. Signaling, rhythmic processes, growth and localization processes had the lowest SSR contents among these transcripts. Similar results were found functional annotation of the date palm sequences containing SSRs [32]. In the cellular component, the majorities of transcripts were assigned with “cell” and “organelle” category, involved in “binding” and “catalytic activity” in the molecular function category. These results suggested that genes that were involved in protein metabolism and biosynthesis were well conserved in plants. The higher occurrence of SSR loci in this ontology level indicated a good potential for using these molecular markers to saturate pathways associated to those functions described above.

In spite of the availability of quite a good number of markers [13], [45], [46], there was lack of dense and saturated genetic map in citrus. Thus, a continuous effort is needed to develop efficient genetic markers for this fruit crop to facilitate the understanding of genetics of complex traits and marker-assisted breeding for development of desired plant types. In this study, we reported identification of a vast number (80,708) of SSR markers in Clementine mandarin. Using 105 randomly selected markers including 78 expressed and 27 genomic SSR markers, we detected 95 (90%) as identifying polymorphism on a panel of 18 citrus cultivars. Unfortunately, seven and four primer pairs fail to transfer in Clementine mandarin and 17 other citrus species, respectively, which might be due to primer (s) across splice sites, the presence of large introns in genomic sequence [33]. Some primer pairs succeed in amplifying in some species while fail in other species, suggesting that null alleles exist. Null alleles might be resulted from some mutations, including the deletion of microsatellites, and indels or substitution in primer binding sites [33]. In addition, the observed sizes of PCR products in 18 species deviate frequently from the expected product sizes according to the transcript sequences. It might be due to the presence of introns in the corresponding genomic regions [33] and/or variation in the repeat numbers. Allelic diversity estimated for these polymorphic markers was an average of 3.85 alleles per locus (range 2–9 alleles). Various genetic parameters viz., allelic diversity, PIC, Ho, He calculated for all the newly developed SSRs demonstrated their utility as genetic markers. Among 70 expressed SSRs, the number of average alleles per locus (N_a_), average heterozygosity (H_O_), and average expected heterozygosity (H_E_) were 4.17, 0.36, and 0. 56, respectively. However, these values were 4.04, 0.20 and 0.57 in genomic SSR. The expressed SSRs revealed high to moderate Na and Ho, which was comparable with genomic SSRs. The expressed SSR markers, in addition to the merits of the genomic SSR markers, were also expected to improve the detection of the marker-trait associations as they were a part of the transcribed domain (s) of the genome. In fact, in recent years emphasis was slowly shifting towards the development of the functional molecular markers instead of the anonymous markers [47]. Thus, they may prove to be more useful for the marker-assisted selection, if found to be associated with a gene/QTL of interest.

## Supporting Information

Table S1 **List of genomic-SSR markers along with their motifs and location in genome.**
(XLS)

Table S2 **List of transcript-SSR markers along with their motifs and location in transcript.**
(XLS)

Table S3 **List of coding sequence-SSR markers along with their motifs and location in gene.**
(XLS)

Table S4 **Characterization of 105 SSR primers in 18 citrus species.** Note: null means numerous non-target bands. ND means not determined.
(XLS)
